# scRDAN: a robust domain adaptation network for cell type annotation across single-cell RNA sequencing data

**DOI:** 10.1093/bib/bbaf344

**Published:** 2025-07-17

**Authors:** Yan Sun, Yan Zhao, Junliang Shang, Baojuan Qin, Xiaohan Zhang, Jin-Xing Liu

**Affiliations:** College of Engineering, Qufu Normal University, No. 80, Yantai Road, Rizhao, 276826, Shandong, China; The School of Computer Science, Qufu Normal University, No. 80, Yantai Road, Rizhao 276826, Shandong, China; The School of Computer Science, Qufu Normal University, No. 80, Yantai Road, Rizhao 276826, Shandong, China; The School of Computer Science, Qufu Normal University, No. 80, Yantai Road, Rizhao 276826, Shandong, China; The School of Computer Science, Qufu Normal University, No. 80, Yantai Road, Rizhao 276826, Shandong, China; The School of Health and Life Sciences, University of Health and Rehabilitation Sciences, No. 369, Dengyun Road, High-tech Zone, Qingdao 266113, Shandong, China

**Keywords:** cell type annotation, batch effects, robustness enhancement strategy, denoising domain adaptation network

## Abstract

Single-cell RNA sequencing technology facilitates the recognition of diverse cell types and subgroups, playing a crucial role in investigating cellular heterogeneity. Cell type annotation, a crucial process in single-cell RNA sequencing analysis, is often influenced by noise and batch effects. To address these challenges, we propose scRDAN, which is a robust domain adaptation network comprising three modules: the denoising domain adaptation module, the fine-grained discrimination module, and the robustness enhancement module. The denoising domain adaptation module mitigates noise interference through feature reconstruction in domains, while leveraging adversarial learning to align data distributions, improving annotation accuracy and robustness against batch effects. The fine-grained discrimination module maintains intra-class compactness and enhances inter-class separability, reducing feature overlap and improving cell type distinction. Finally, the robustness enhancement module introduces noise from various perspectives in both domains, enhancing robustness and generalization. We evaluate scRDAN on simulated, cross-platforms, and cross-species datasets, comparing it with advanced methods. Results demonstrate that scRDAN outperforms existing methods in handling batch effects and cell type annotation.

## Introduction

Single-cell RNA sequencing (RNA-seq) technology allows for the accurate profiling of gene expression variability at the single-cell level, revealing differences between distinct cell populations within the same tissue. Therefore, understanding cellular heterogeneity is critical for diagnosis and treatment. Cell type annotation is a key process in understanding cellular heterogeneity [1], as it involves identifying different cell types, exploring interactions between cells, and elucidating their roles in specific biological processes. This process is of great significance for advancing biomedical research [2].

Currently, cell type annotation approaches are primarily classified into two types: conventional manual annotation methods and automated annotation methods. Traditional manual cell type annotation relies on expert knowledge, where cell types are annotated by analyzing cellular morphological features and gene expression patterns. Although this method offers high accuracy and a deep understanding of specific cell types, allowing the capture of complex biological information, it has several drawbacks, including being time-consuming, highly subjective, and dependent on expert knowledge. Additionally, traditional manual cell type annotation is inefficient when processing large amounts of data, making it unsuitable for large-scale applications. To address these challenges, automated cell type annotation methods have emerged. Similarity-based cell type annotation methods, such as scmap [3], SingleR [4], and CelliD [5], accomplish cell type annotation task by calculating the similarity between unlabeled target domain cells and labeled source domain cells. These methods are efficient but face challenges such as dependency on similarity measures, susceptibility to noise, and limited ability to handle nonlinear relationships. Machine learning-based cell type annotation methods, such as Seurat [6], SingleCellNet [7], and scClassify [8], predict cell types by leveraging labeled source domain data to train models that capture the relationship between cell types and their corresponding features. While these methods offer improved cell type annotation performance, they are highly dependent on high-quality input data and lack mechanisms specifically designed to address batch effects [9], which can affect the overall cell type annotation performance.

Deep learning-based cell type annotation methods [10, 11], such as CIForm [12], SCLSC [13], and scGAD [14], utilize deep learning models to automatically extract complex features from high-dimensional data in the source domain and capture intricate nonlinear relationships between cells, enabling accuracy. Early deep learning methods primarily focus on using neural networks to extract cell type features, thereby improving the accuracy of cell type annotation. However, with the advancement of technology and the increasing scale of data, the issue of batch effects has gradually emerged. Batch effects refer to systematic biases introduced by non-biological factors such as experimental conditions, equipment, and sequencing batches, which cause variations in the performance of the same cell type across different batches or platforms. This bias significantly affects the generalization ability of the models, particularly when applied across platforms or species. In early deep learning models, batch effects are often overlooked, leading to excellent performance on source domain datasets but poor performance when applied to different target domain datasets. In recent years, with growing attention to batch effects, various models specifically designed to handle batch effects have been developed, such as Harmony [15], LIGER [16], BEENE [17], and DB-AAE [18]. These models use specific strategies to help models better adapt to data from different sources. However, these methods primarily focus on addressing batch effects, highlighting the importance of developing cell type annotation models that effectively mitigate batch effects.

With the advancement of deep learning technologies and a deeper understanding of batch effects, domain adaptation-based cell type annotation methods have gradually emerged as effective solutions. These methods, such as scAdapt [19], scGCN [20], scDOT [21], and SCdenoise [22], employ strategies such as domain adaptation or metric learning to address batch effects, thereby improving accuracy. Although these methods perform excellently in alleviating batch effects, many models still primarily focus on domain global alignment. This domain global alignment may lead to blurred cell type boundaries, thus affecting accuracy. Therefore, developing cell type annotation models capable of effectively handling batch effects while maintaining clear cell type boundaries is essential.

Considering the issues of noise, batch effects, and the potential blurring of cell type boundaries, a robust domain adaptation network (scRDAN) is proposed. The denoising domain adaptation module mitigates the impact of noise interference and batch effects on the cell type annotation task by reconstructing domain-specific features and achieving distribution alignment through adversarial learning. The fine-grained discrimination module ensures intra-class compactness while increasing inter-class separability. By minimizing feature overlap, it improves the distinctiveness of different cell types, resulting in improved cell type annotations. The robustness enhancement module introduces controlled noise from multiple perspectives across both domains, reinforcing the stability and boosting its generalization ability of scRDAN across diverse datasets. The experimental results indicate that scRDAN achieves superior performance compared with other methods in both cell type annotation and batch effect correction.

## Methods and datasets

### The overview of scRDAN

**Figure 1:**
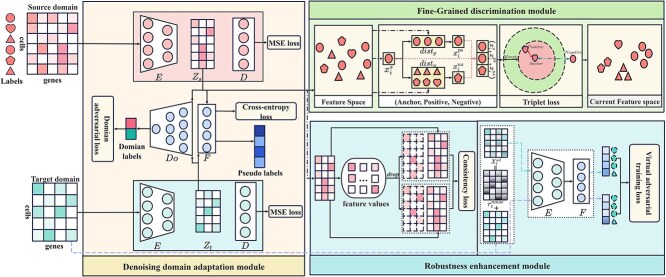
Overview of scRDAN.

The overview of scRDAN is illustrated in Fig. 1. Based on its network architecture, it includes an encoder, a decoder, a domain discriminator, and a label classifier.

#### Denoising domain adaptation module

Considering noise and batch effects, directly performing cell type annotation on target domain data may result in poor performance. Firstly, noise can obscure the true feature information, reducing cell type annotation effectiveness, while batch effects introduce systematic biases, leading to inaccurate cell type identification. Transferring cell type information from source to target domain can be challenging, affecting generalization ability. Target domain data without denoising and batch correction may lower cell type annotation accuracy and the reliability of biological interpretation. Therefore, denoising and batch effect correction are crucial steps to ensure successful annotation.

For denoising task, encoder $E(\cdot )$ takes the gene expression matrix ${{X}_{s}}=\{x_{1}^{s},\ldots ,x_{{{M}_{s}}}^{s}\}\in{{R}^{{{M}_{s}}\times n}}$ of source domain and ${{X}_{t}}=\{x_{1}^{t},\ldots ,x_{{{M}_{t}}}^{t}\}\in{{R}^{{{M}_{t}}\times n}}$ of target domain as input and learns the latent feature representation. Here, $n$ denotes the number of highly expressed genes selected, which is 2000. During the reconstruction phase, decoder $D(\cdot )$ effectively filters out noise and extracts more relevant features, thereby restoring the true biological signals.

The denoising goal is achieved by minimizing the difference between original and reconstructed gene expression matrix. The reconstruction loss ${{L}_{mse}}$ is defined as follows:


(1) \begin{align*}& {{L}_{mse}}=\frac{1}{{{M}_{s}}}\sum\limits_{i=1}^{{{M}_{s}}}{{{(x_{i}^{s}-\overline{x}_{i}^{s})}^{2}}}+\frac{1}{{{M}_{t}}}\sum\limits_{j=1}^{{{M}_{t}}}{{{(x_{j}^{t}-\overline{x}_{j}^{t})}^{2}}},\end{align*}

where ${{M}_{s}}$ denotes the number of cells in source domain, $x_{i}^{s}$ denotes the feature vector of the cell $i$ in the source domain, and $\bar{x}_{i}^{s}$ represents reconstructed feature representation of the corresponding cell $i$. ${{M}_{t}}$ refers to the number of cells in target domain, $x_{j}^{t}$ denotes feature representation of the cell $j$ in target domain, and $\bar{x}_{j}^{t}$ represents the reconstructed feature representation of the corresponding cell $j$.

To address batch effects, an adversarial relationship is established to alleviate the distribution discrepancy between two domains. Specifically, the gene expression matrix ${{X}_{s}}$ of the source domain, true cell labels, and gene expression matrix ${{X}_{t}}$ of the target domain are used as inputs. $E(\cdot )$ is employed to extract the corresponding feature representations ${{Z}_{s}}=E({{X}_{s}})$ and ${{Z}_{t}}=E({{X}_{t}})$ for two domains, respectively, and map them to a lower dimensional feature space. After feature extraction, ${{Z}_{s}}$ and ${{Z}_{t}}$ are fed into the domain discriminator $Do(\cdot )$ to determine whether the input data originate from the source or target domain. Specifically, $E(\cdot )$ aims to generate feature representations that confuse $Do(\cdot )$, while $Do(\cdot )$ strives to improve its accuracy in domain classification. By optimizing this adversarial relationship, the model’s performance on the cell type annotation task is enhanced, and its generalization ability on the target domain is significantly improved. This process effectively mitigates the influence of batch effects, providing strong support for accurate cell type identification.

The label predictor $F(\cdot )$ continuously trains and optimizes based on the features extracted by $E(\cdot )$, aiming to accurately predict the cell labels. Reducing the disparity between true labels and predicted probabilities enhances accuracy. The label prediction loss ${{{L}}_{CE}}$ is defined as follows:


(2) \begin{align*}& {{{L}}_{CE}}=-\frac{1}{{{M}_{s}}}\sum\limits_{i=1}^{{{M}_{s}}}{\sum\limits_{c=1}^{K}{y_{i,c}^{s}}}\log ({{p}_{i,c}}),\end{align*}

where, if the label of cell $i$ is correct, $y_{i,c}^{s}$ takes the value of 1; otherwise, $y_{i,c}^{s}$ is assigned a value of 0. $K$ represents the number of cell types, and ${{p}_{i,c}}$ denotes the predicted probability of cell $i$ belonging to cell type $c$.

$Do(\cdot )$ is trained to accurately distinguish the domain origin of the data based on the feature representations learned by $E(\cdot )$. By continuously minimizing the difference between true domain value and predicted domain value, the domain discrimination accuracy is improved. The domain discrimination loss $L_{DA}$ is implemented as follows:


(3) \begin{align*} \begin{split} L_{DA} &= -\frac{1}{M_{s}} \sum_{i=1}^{M_{s}} \log \left( Do(G(x_{i}^{s})) \right) \\ &\quad - \frac{1}{M_{t}} \sum_{j=1}^{M_{t}} \log \left( 1 - Do(G(x_{j}^{t})) \right) \end{split}\end{align*}

To establish the adversarial relationship between $E(\cdot )$ and $Do(\cdot )$, the domain discrimination loss needs to be maximized. The Gradient Reversal Layer (GRL) is inserted between $E(\cdot )$ and $Do(\cdot )$ to facilitate the learning of domain-invariant representations. Specifically, during the forward pass, the GRL acts as an identity function, directly passing the features extracted by $E(\cdot )$ to $Do(\cdot )$. During the backward pass, however, it multiplies the incoming gradients by a negative constant, effectively reversing their direction. This mechanism encourages $E(\cdot )$ to learn feature representations that are indistinguishable to $E(\cdot )$, thereby aligning the feature distributions of the source and target domains in the latent space.

The total loss ${{L}_{DDANN}}$ of denoising domain adaptation module is as follows:


(4) \begin{align*}& {{L}_{DDANN}}={{L}_{CE}}+{{\lambda} _{DA}}{{L}_{DA}}+{{\lambda} _{mse}}{{L}_{mse}},\end{align*}

where ${{\lambda }_{DA}}$ and ${{\lambda }_{mse}}$ represent the weight coefficients corresponding to each loss.

#### Fine-grained discrimination module

The denoising domain adaptation module may focus too much on aligning global domain distributions, neglecting local details crucial for cell type identification. If these features are lost during alignment, accuracy may suffer. Effective annotation requires both global feature alignment and capturing subtle differences between cell types. Thus, a fine-grained discrimination module is necessary to enhance clustering between anchor and negative cells while minimizing distance to positive cells, improving scRDAN’s ability to differentiate cell types and boosting annotation accuracy.

Specifically, the distance between different feature vectors is first calculated to construct the corresponding distance matrix $dist\in{{\text{R}}^{M_{s}\times M_{s}}}$. Based on the label information of the source domain cells, cells are sequentially chosen as the anchor cell $x_{i}^{s}$ according to a predetermined order. For each anchor cell, the following operations are performed: based on $x_{i}^{s}$, the distance matrix $dist$ is first used to determine the distance set $dis{{t}_{p}}$ for same-type cells and the distance set $dis{{t}_{n}}$ for different-type cells. Then, the farthest positive cell $x_{i}^{ps}$ from the anchor cell is selected from $dis{{t}_{p}}$, while the closest negative cell $x_{i}^{ns}$ to the anchor cell is selected from $dis{{t}_{n}}$. This ensures that the distance between the positive cell is minimized, while the distance between the anchor cell and the negative cell is maximized. This process focuses on the local differences between cells, thereby maintaining the distinctiveness between cell types. This method not only reduces the distribution difference during the global alignment process but also avoids the problem of fuzzy cell type distinction during alignment, thus effectively improving performance.

By continuously minimizing the distance between $x_{i}^{s}$ and $x_{i}^{ps}$, while maximizing the distance between $x_{i}^{s}$ and $x_{i}^{ns}$, the discriminative ability between features of different cell types is enhanced. The fine-grained discrimination loss ${L}_{tri}$ is as follows:


(5) \begin{align*}& {{L}_{tri}}=\sum\limits_{\text{i}=1}^{{{\text{M}}_{\text{s}}}}{{{[||E(x_{i}^{s})-E(x_{i}^{ps})|{{|}^{2}}-||E(x_{i}^{s})-E(x_{i}^{ns})|{{|}^{2}}+\alpha ]}_{+}}},\end{align*}

where $E(x_{i}^{s})$ represents the feature representation of $x_{i}^{s}$, $E(x_{i}^{ps})$ represents the feature representation of $x_{i}^{ps}$ that is similar to $x_{i}^{s}$, $E(x_{i}^{ns})$ represents the feature representation of $x_{i}^{ns}$ that is dissimilar to $x_{i}^{s}$, and $\alpha $ is the hyperparameter of the margin, ensuring that the distance between $x_{i}^{ns}$ and $x_{i}^{s}$ is at least $\alpha $ greater than the distance to $x_{i}^{ps}$. ${{[\cdot ]}_{+}}$ denotes the positive function, which takes the value inside the parentheses if it is greater than 0, otherwise it takes 0.

#### Robustness enhancement module

Although feature reconstruction helps recover original features from noise, the model can still be affected by noise. To enhance robustness, this study introduces a robustness enhancement module that improves adaptability to noise and data variations while maintaining feature quality. Comprising virtual adversarial training loss and consistency loss, the module boosts noise resistance by introducing noise into both source and target domain features, thereby improving annotation accuracy and reliability.

Firstly, a consistency strategy is applied to the feature representations of the source domain data by randomly dropping a subset of features to generate two sets of augmented features. By comparing the differences between the source feature and each augmented feature, more robust feature representations are learned, thereby reducing reliance on local features. This strategy enhances adaptability to data variations by continually minimizing the gap between the source feature and augmented features, effectively improving robustness and generalization ability in noisy environments.

Specifically, a random dropout operation is applied to the feature $x_{i}^{s}$ in the source domain to construct multiple augmented features ${x^{\prime}}_{i1}^{s}$ and ${x^{\prime}}_{i2}^{s}$. Then, the feature pairs are formed by pairing $x_{i}^{s}$ with ${x^{\prime}}_{i1}^{s}$ and $x_{i}^{s}$ with ${x^{\prime}}_{i2}^{s}$, and the consistency loss is applied to ensure consistency between feature pairs. This process is repeatedly executed to ensure that each cell undergoes the described operation. Introducing randomness helps scRDAN more effectively learn the underlying relationships between features, thereby enhancing its robustness.

By continuously reducing the gap between the source feature and its corresponding augmented features, overall robustness is ensured. The consistency loss ${L}_{con}$ is as follows:


(6) \begin{align*}& {{L}_{con}}=\frac{1}{{{M}_{s}}}\sum\limits_{i=1}^{{{M}_{s}}}{[||E(x_{i}^{s})}-E({x^{\prime}}_{i1}^{s})|{{|}^{2}}+||E(x_{i}^{s})-E({x^{\prime}}_{i2}^{s})|{{|}^{2}}]\end{align*}

Additionally, a virtual adversarial training strategy adds random perturbations to the target domain’s cell feature representations. This improves robustness and generalization by reducing reliance on data distribution and enhancing resistance to noise. The strategy mitigates potential noise interference in the target domain, ensuring robustness in practical applications and improving the ability to handle complex biological data.

Specifically, $x_{i}^{t}$ of each cell in the target domain data is determined. Then, random perturbations are generated as required, and the maximum perturbation $r_{i}^{t{\_}noise}$ is selected to create the corresponding adversarial feature vector $x_{i}^{ad}=x_{i}^{t}+r_{i}^{t{\_}noise}$ for that cell. This adversarial feature vector is input into encoder for feature extraction, followed by prediction using the label classifier to obtain the corresponding cell type probability distribution. For each cell in the target domain, this process is repeated to generate the adversarial feature vector, resulting in a set of overall adversarial features $X_{t}^{ad}={{X}_{t}}+r_{t}^{noise}=\{x_{0}^{ad},\ldots ,x_{{{M}_{t}}}^{ad}\}$ for the target domain. Next, the overall probability distribution $p({{Y}_{t}}|{{X}_{t}},\theta )$ of the target domain data and the corresponding adversarial probability distribution $p({{Y}_{t}}|X_{t}^{ad},\theta )$ are computed.

By minimizing the difference between $p({{Y}_{t}}|{{X}_{t}},\theta )$ and $p({{Y}_{t}}|X_{t}^{ad},\theta )$, scRDAN is trained to withstand perturbations, thereby improving robustness. The virtual adversarial training loss ${{L}_{vat}}$ is defined as follows:


(7) \begin{align*}& {{L}_{vat}}({{X}_{t}},\theta) ={{D}_{KL}}[p({{Y}_{t}}|{{X}_{t}},\theta ),p({{Y}_{t}}|X_{t}^{t{\_}ad},\theta)],\end{align*}

where $\theta $ represents the model parameters, and ${{D}_{KL}}(\cdot )$ denotes KL divergence.

The total loss ${{L}_{\text{CV}}}$ of robustness enhancement module is as follows:


(8) \begin{align*}& {{L}_{\text{CV}}}={{L}_{con}}+{{\lambda} _{vat}}{{L}_{vat}},\end{align*}

where ${{\lambda } _{vat}}$ represents the weight coefficient of the corresponding loss, typically set to 1.0.

The total loss ${{L}_{total}}$ of scRDAN consists of ${{L}_{DDANN}}$, ${{L}_{tri}}$, and ${{L}_{CV}}$.


(9) \begin{align*}& {{L}_{total}}={{L}_{DDANN}}+{{L}_{tri}}+{{L}_{CV}}\end{align*}

### Datasets and preprocessing

Simulated datasets are generated using the R package Splatter with similar cell type compositions across batches. Two datasets are simulated: the source domain with 2000 cells and the target domain with 1000 cells, each containing 10 000 genes. Cells are divided into four groups representing different cell types. Batch effects are introduced by setting the batch parameters batch.facLoc and batch.facScale to $\{0.2,0.4,0.6,0.8,1.0,1.2,1.4\}$, with higher values indicating stronger effects. Clustering difficulty is adjusted by setting de.fracScale to 0.2 for weak signals. Simulations are repeated five times for each batch effect intensity, and the average result is used.

The cross-platforms datasets PbmcBench contains single-cell RNA-seq data of human peripheral blood mononuclear cells [23] from multiple sequencing platforms. Specifically, the datasets are from 10x Chromium (v2) and 10x Chromium (v3) [24] platforms, CEL-Seq2 [25], Drop-seq [25], inDrops [26], Seq-Well [27], and Smart-seq2 [28]. These datasets are utilized to assess and contrast the performance of various platforms in cell type annotation, helping to address technical differences and batch effects.

The cross-species datasets include pancreatic data from both humans and mice. Specifically, mouse pancreatic data are sourced from Baron mouse datasets [29], while human pancreatic data come from multiple sources, including Baron human [29], Segerstolpe [30], Wang [31], Xin [32], and Muraro [33]. To facilitate gene name conversion between humans and mice, the homologous gene table provided by SingleCellNet is used, keeping only the genes common to both species.

Data preprocessing is crucial in sequencing analysis, with the primary aim of improving data quality and usability. Selecting highly variable genes allows for the effective capture of biological differences between cells, improving the accuracy of cell type annotation.

For simulated datasets, the gene expression matrix is normalized using the TPM (Transcripts Per Million) to eliminate differences in sequencing depth and gene length across samples, thereby enhancing data comparability. TPM standardizes the raw expression levels of genes by adjusting for gene length and converts them to expression levels per million, ensuring that the total expression across different samples is consistent. The use of TPM in simulated datasets provides a consistent baseline for gene expression patterns under different conditions, supporting evaluation and validation.

For real datasets, the gene expression matrix is first normalized using NormalizeData in Seurat. This function adjusts the gene expression values of different cells to a consistent scale through log-transformation, with a scaling factor of 10 000, thus minimizing the impact of technical variations. FindVariableGenes was then employed to select the top 2000 highly variable genes, which show significant differences across cell types and better capture biological variations, enhancing the efficiency of subsequent cell type annotation.

### Hyperparameter settings

scRDAN is implemented in PyTorch. During training, we use an inverse decay learning rate schedule, where the learning rate decreases inversely with the number of epochs, helping the model converge more stably in later stages. The starting learning rate is set to 0.001, with a decay factor of 0.001 and a power exponent of 0.9. Stochastic Gradient Descent is employed to minimize the loss function and update the parameters, using a learning rate of 0.001, weight decay of $5e-4$, and momentum of 0.9. Other parameters are adjusted based on dataset characteristics.

### Evaluation metrics

Cell type annotation performance is assessed using accuracy, a metric that varies between 0 and 1. A higher accuracy value indicates better precision in annotating cell types.


(10) \begin{align*}& accurac{{y}_{c}}=\frac{predic{{t}_{c}}}{tru{{e}_{c}}},\end{align*}

where $accurac{{y}_{c}}$ indicates annotation accuracy of cell type $c$, $predic{{t}_{c}}$ denotes number of cells correctly predicted to cell type $c$, and $tru{{e}_{c}}$ represents number of cells with real cell type $c$.


(11) \begin{align*}& Accuracy=\frac{\sum\limits_{c=1}^{K}{accurac{{y}_{c}}}}{K},\end{align*}

where $Accuracy$ represents the mean accuracy of the annotation across all cell types.

Additionally, Silhouette score is used to evaluate batch effect correction performance. The score ranges from −1 to 1, where values closer to 1 suggest better batch correction and values near −1 indicate worse performance. Silhouette score is used to assess batch effect correction performance. The score ranges from −1 to 1, with values closer to 1 indicating better batch correction and values closer to −1 indicating poorer performance.


(12) \begin{align*}& a(i)=\frac{1}{|{{C}_{i}}|-1}\sum\limits_{j\in{{C}_{i}},j\ne i}{d(i,j)},\end{align*}

where ${{C}_{i}}$ is the cluster to which cell $i$ belongs, and $d(i,j)$ is the distance between cell $i$ and cell $j$.


(13) \begin{align*}& b(i)=\underset{{{C}_{k}}\ne{{C}_{i}}}{\mathop{\min }}\,\frac{1}{|{{C}_{k}}|}\sum\limits_{j\in{{C}_{k}}}{d(i,j)},\end{align*}

where ${{C}_{k}}$ is a cluster different from ${{C}_{i}}$.


(14) \begin{align*}& s(i)=\frac{b(i)-a(i)}{\max (a(i),b(i))},\end{align*}

where $a(i)$ denotes the average distance between cell $i$ and all other cells within the same cluster, and $b(i)$ represents the average distance between cell $i$ and all cells in the nearest neighboring cluster, which is the closest cluster to cell $i$.


(15) \begin{align*}& \begin{matrix} Silhouette&score \\ \end{matrix}=\frac{1}{{{M}_{s}}+{{M}_{t}}}\sum\limits_{i=1}^{{{M}_{s}}+{{M}_{t}}}{s(i)}\end{align*}

### Experimental results on simulated datasets

**Figure 2:**
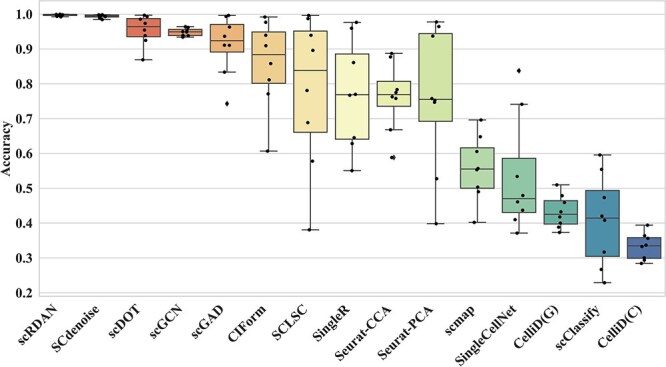
Comparison of Accuracy of scRDAN and other methods under different batch effect intensities.

Figure 2 illustrates the comparison of cell type annotation performance between scRDAN and other methods under varying batch effect intensities. Overall analysis indicates that as the batch effect intensity increases, the performance of all methods declines, highlighting the significant impact of batch effects on the Accuracy.

scRDAN maintains stable performance as batch effect intensity increases (from 0.2 to 1.4), outperforming all comparison methods and effectively mitigating batch effects. SCdenoise ranks second, maintaining stable performance (0.985) even at the highest batch effect intensity. Deep learning-based methods like scDOT, scGCN, scGAD, CIForm, and SCLSC also perform well, capturing deep features and ensuring robustness against batch effects. In contrast, methods like SingleR, Seurat-CCA, Seurat-PCA, and scmap show weaker performance in handling batch effects. Among Seurat-CCA and Seurat-PCA, Seurat-CCA outperforms Seurat-PCA with better accuracy and stability, despite Seurat-PCA excelling on some datasets. CelliD(C) and CelliD(G) show average annotation performance.

### Experimental results on cross-platforms datasets

**Figure 3:**
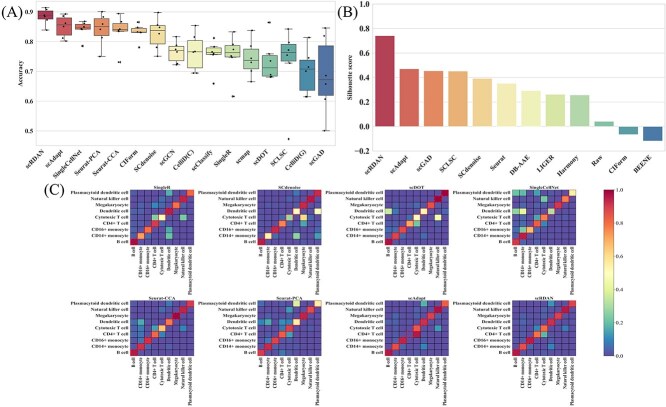
(A) Accuracy Comparison of of scRDAN and other methods on cross-platforms datasets. (B) Silhouette Score Comparison between scRDAN and other methods on cross-platform datasets. (C) Comparison of cell type annotation ability of scRDAN and other methods on 10x_inDrops.

Figure 3(A) and Fig. 3(B) present the performance of scRDAN and other methods in terms of Accuracy and Silhouette scores on cross-platforms datasets. For cell type annotation methods with batch effect mitigation capabilities, such as scRDAN, scAdapt, and scGAD, the overall analysis shows that these methods effectively alleviate the impact of batch effects and maintain high accuracy in cell type annotation tasks. This result further validates that effectively handling batch effects can significantly improve cell type annotation outcomes.

As shown in Fig. 3(A), scRDAN outperforms all other methods in Accuracy. This superior performance is primarily attributed to the effective collaboration between its denoising domain adaptation module and fine-grained discriminative module. Specifically, the denoising domain adaptation module ensures the restoration of the original feature information through feature reconstruction and achieves domain global alignment through adversarial learning, effectively mitigating the impact of batch effects on annotation. Meanwhile, the fine-grained discriminative module optimizes the positional relationships of cell types in the feature space, enhancing the discriminability between cells and further improving the accuracy. In contrast, scAdapt ranks second overall. scAdapt effectively alleviates batch effects through its virtual adversarial domain adaptation method, ensuring high accuracy in cell type annotation. Compared with Seurat-CCA, Seurat-PCA demonstrates more stable overall performance on cross-platforms datasets, exhibiting stronger robustness, whereas Seurat-CCA shows some variability when handling data discrepancies. Additionally, other methods like scGCN and scClassify also exhibit relatively stable overall cell type annotation performance.

As shown in Fig. 3(B), scRDAN achieves Silhouette score of 0.742 on the cross-platforms datasets, ranking first. This indicates that scRDAN exhibits excellent batch correction ability and effectively alleviates batch effects. This outstanding performance is primarily attributed to the fine-grained discrimination module, which effectively regulates the positional relationships among cells of the same and different types. By ensuring clear distinctions between adjacent cell types, the module alleviates the issue of blurred cell-type boundaries and thereby effectively eliminates systematic biases introduced by technical variations. In comparison, methods such as scAdapt, scGAD, and SCLSC have similar Silhouette scores, suggesting that they possess comparable abilities in batch effect mitigation. On the other hand, Silhouette scores of CIForm and BEENE, two batch correction methods, are negative and lower than those of the raw data (RAW), likely due to insufficient batch correction. Furthermore, these methods are more sensitive to noise in high-dimensional data, which may lead to the loss of some biological signals and weaken their performance in batch correction tasks.

As shown in Fig. 3(C), the heatmap provides a more intuitive representation of the accuracy and error rates of each model in cell type annotation, clearly reflecting the performance differences of various models across different cell types. The dashed diagonal line in the figure represents correct cell type annotations, while the off-diagonal sections indicate incorrect cell type annotations. The color gradient represents the accuracy or error rate of the cell type annotation. Specifically, SingleR misclassifies some Plasmacytoid dendritic cell, CD16+ monocyte, and CD14+ monocyte as Dendritic cell, and some Cytotoxic T cell as Natural killer cell. SCdenoise misclassifies some Plasmacytoid dendritic cell and CD14+ monocyte as Dendritic cell, and some Dendritic cell and CD14+ monocyte as Plasmacytoid dendritic cell. scDOT misclassifies some Dendritic cell as B cell, and some CD4+ T cell as Cytotoxic T cell. SingleCellNet’s misclassifications are more complex, with some Plasmacytoid dendritic cell misclassified as B cell, CD14+ monocyte, and CD4+ T cell, some Natural killer cell misclassified as Cytotoxic T cell, some Dendritic cell misclassified as B cell and CD14+ monocyte, and some CD4+ T cell misclassified as Cytotoxic T cell. Seurat-CCA shows more prominent errors, misclassifying multiple cell types as CD14+ monocyte and Cytotoxic T cell. Seurat-PCA misclassifies Plasmacytoid dendritic cell, Dendritic cell, and other cell types as various other cell types, resulting in an overall moderate accuracy. In contrast, scAdapt has fewer errors, but the most notable one is misclassifying most CD4+ T cell as Cytotoxic T cell. scRDAN annotates nearly all CD4+ T cell correctly and is able to resolve the misclassifications of other models, such as those of Plasmacytoid dendritic cell, Natural killer cell, and Dendritic cell. The overall experimental results demonstrate that scRDAN, through its denoising domain adaptation module and fine-grained discriminative module, effectively aligns cells at both the domain and cell levels, addressing cross-platforms data distribution discrepancies and successfully transferring cell labels from source to target domain, thereby achieving accurate cell type annotation.

**Figure 4:**
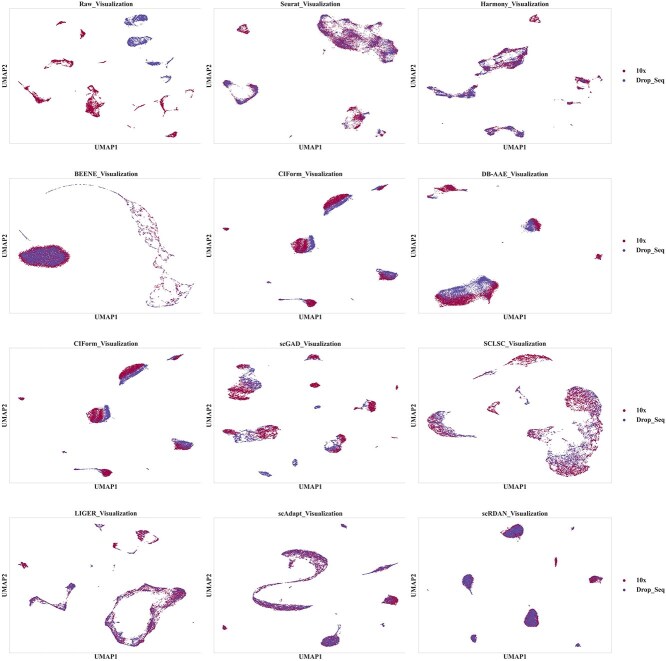
UMAP visualization results by batches on 10x_Drop-seq.

**Figure 5:**
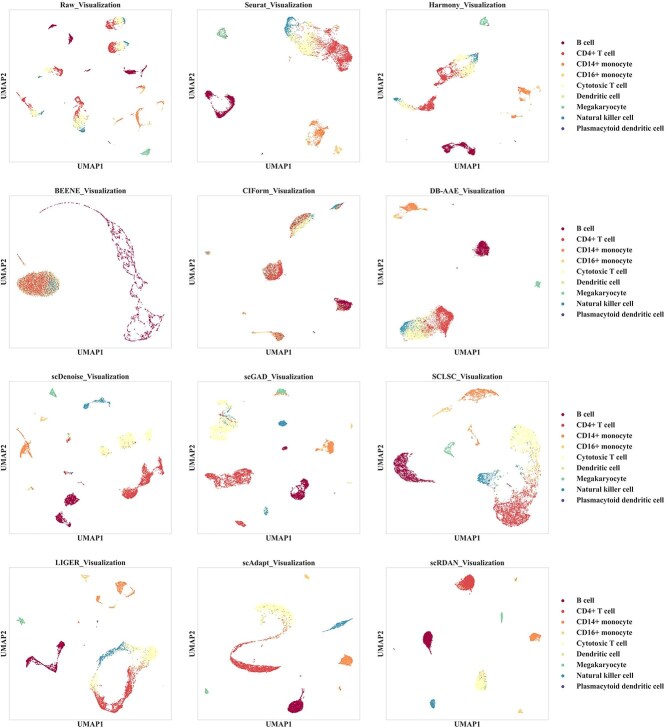
UMAP visualization results by cell types on 10x_Drop-seq.

Figure 4 and Fig. 5 show the comparative visualization results of each model on the 10x_Drop-seq cross-platforms dataset, grouped by batch and cell type. Firstly, the UMAP visualization results by batches indicate that the source and the target domain data are relatively well integrated, with scRDAN performing the best in terms of mitigating batch effects. Secondly, the UMAP visualization results by cell types show that scRDAN effectively separates different cell types, with cells of the same type tightly clustered together, enhancing the discriminability between cell types and enhancing the Accuracy. In contrast, other methods exhibit some degree of mixing between different cell types, especially between functionally similar B cells, cytotoxic T cells, and natural killer cells, with the mixing problem being more prominent in these cases. The UMAP visualization results further indicate that it effectively reduces batch effects and maintains clear boundaries between cell types, highlighting its advantage in data integration.

### Experimental results on cross-species datasets

**Figure 6:**
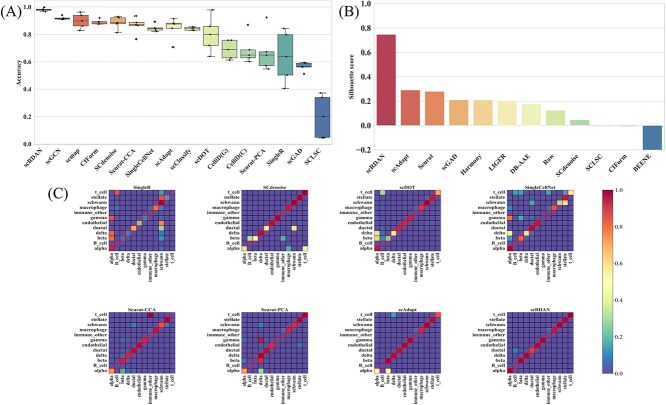
(A) Accuracy Comparison of scRDAN and other methods on cross-species datasets. (B) Silhouette Score Comparison of scRDAN and other methods on cross-species datasets. (C) Comparison of cell type annotation capabilities of scRDAN and other comparison methods on Baron_mouse_combination.

As depicted in Fig. 6(A), scRDAN achieves the highest performance in the cell type annotation task on cross-species datasets. The denoising domain alignment module and fine-grained discriminative module effectively mitigate the noise in the data, identify potential biological variations, and successfully perform cross-species cell labels transfer. scGCN ranks second overall, capturing structured information between cells through graph convolutional networks and extracting cross-species shared features, thereby enhancing the model’s robustness to noise. Methods such as scmap, CIForm, and SCdenoise show similar annotation performance on the cross-species datasets. The performance difference between Seurat-CCA and Seurat-PCA is more pronounced due to their distinct feature integration approaches. Seurat-CCA utilizes canonical correlation analysis (CCA) to identify and align cross-species shared features, effectively reducing the impact of batch effects on annotation accuracy. In contrast, Seurat-PCA, based on principal component analysis (PCA), is more suitable for single datasets and struggles with cross-species feature differences, making it more susceptible to batch effects. Therefore, Seurat-CCA outperforms Seurat-PCA on cross-species datasets. In comparison, SCLSC performs poorly on the cross-species datasets, possibly due to its contrastive learning approach, which is less effective in extracting cross-species shared features, making it more sensitive to batch effects. Additionally, its reliance on supervised learning with labels makes it vulnerable to label shifts or inconsistencies, further reducing annotation accuracy.

As shown in Fig. 6(B), scRDAN achieves the Silhouette score of 0.737 on cross-species datasets, ranking first. This result indicates that scRDAN exhibits a significant advantage in handling cross-species datasets, particularly demonstrating higher accuracy and stability in distinguishing data distributions and cell type boundaries. The fine-grained discrimination module minimizes the distance between anchor and positive cells while maximizing the distance to negative cells, promoting the aggregation of cells of the same type and the dispersion of different types in the low-dimensional space. This enhances the clarity of cell type boundaries and effectively mitigates the interference caused by cross-species distribution discrepancies. In comparison, methods such as scAdapt, Seurat, and scGAD show similar Silhouette scores, suggesting comparable capabilities in handling batch effects. However, batch correction methods like SCLSC, CIForm, and BEENE have negative Silhouette scores, which are lower than the Silhouette score of the raw data (RAW). This may be due to shortcomings in the batch correction processes, where they fail to effectively capture inter-species biological differences. Additionally, there is insufficient robustness in handling noise and variability across different batches, resulting in poorer performance than the raw data.

As shown in Fig. 6(C), the heatmap of the Baron_mouse_ combination dataset illustrates the performance of various models in the cell type annotation task. SingleR misclassifies some beta, delta, and gamma as alpha. SCdenoise misclassifies beta as B_cell and immune_other, alpha as macrophage and t_cell, and ductal as schwann. scDOT misclassifies multiple cell types, including delta and beta, as alpha, and some beta as B_cell. SingleCellNet misclassifies t_cell as B_cell, beta and other cell types, and misclassifies macrophage, gamma, and other cell types as alpha. Seurat-CCA misclassifies most t_cell as immune_other, while Seurat-PCA misclassifies most gamma as delta. scAdapt misclassifies a few t_cell as delta and some alpha as beta. In contrast, scRDAN achieves near-perfect annotation, with only a few ductal cells misclassified as B_cell. These results indicate that scRDAN can effectively manage biological noise across species while preserving inter-species heterogeneity, allowing for accurate cell type annotation.

**Figure 7:**
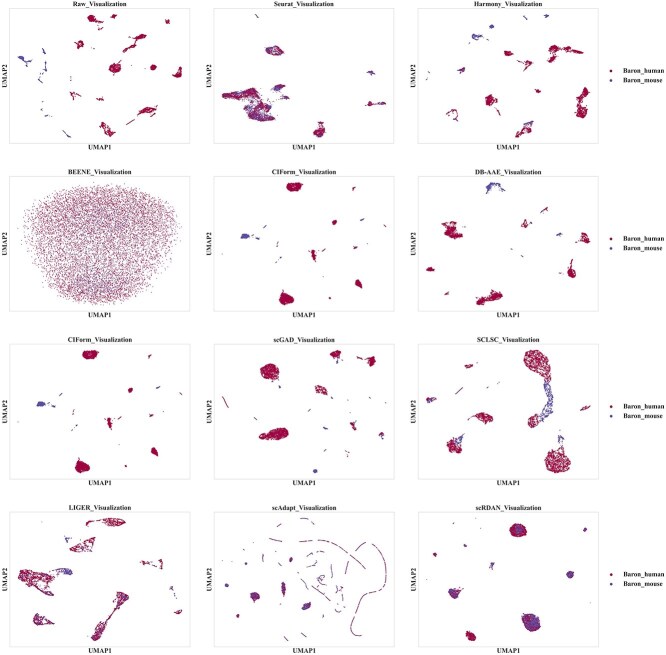
UMAP visualization results by batches on Baron_human_Baron_mouse.

**Figure 8:**
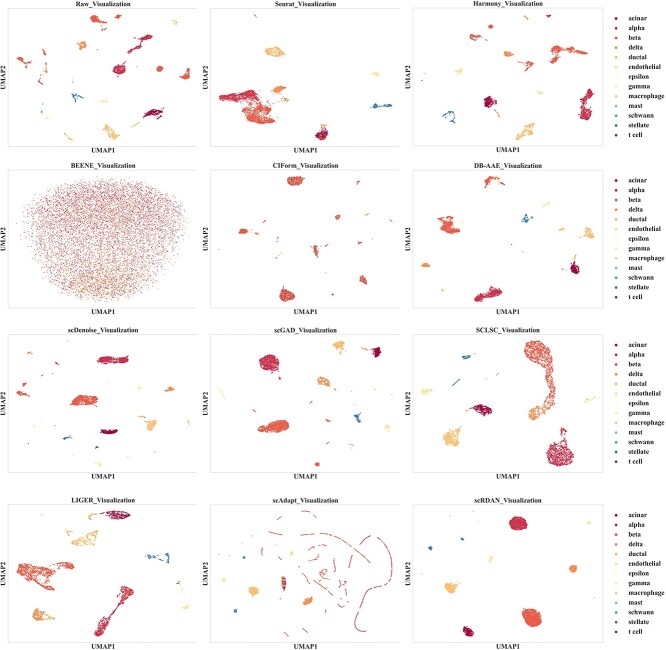
UMAP visualization results by cell types on Baron_human_Baron_mouse.

The visualization results on the cross-species datasets Baron_human_Baron_mouse by batches and cell type, are shown in Fig. 7 and Fig. 8, respectively. Firstly, the UMAP visualization results by batches demonstrate that scRDAN achieves the best alignment between the source and target domain, enabling domain global integration of both domains and effectively alleviating the influence of batch effects on cell type annotation. Secondly, the UMAP visualization results by cell types show that scRDAN clearly separates different cell types, with similar cell types tightly clustered together, thus avoiding potential issues of blurred cell boundaries during domain global alignment. This enhances the distinguishability of cell types and improves cell type annotation accuracy. In contrast, other methods exhibit noticeable mixing between different cell types, particularly between similar cell types such as Cytotoxic T cell and CD4+ T cell, leading to more frequent misclassification. Moreover, scRDAN captures the differences and commonalities in gene expression across species, assisting in learning more generalized feature representations, thus enhancing the accuracy of cell type annotation.

### Ablation experiments

**Figure 9:**
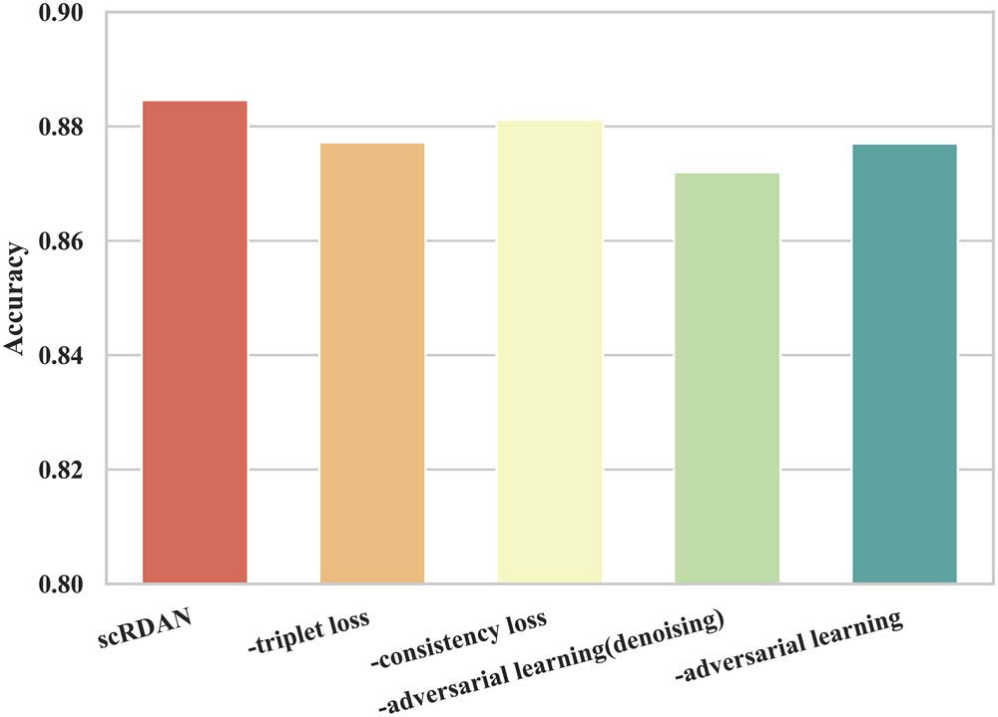
Comparison of components ablation results under Accuracy on the cross-platforms datasets.

**Figure 10:**
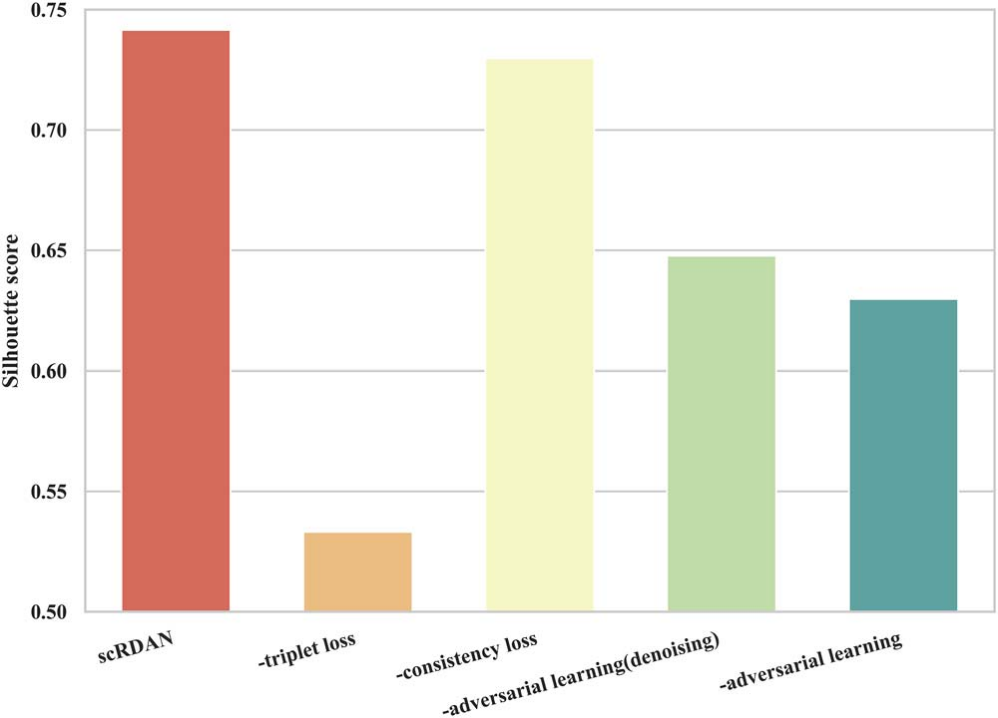
Comparison of components ablation results under Silhouette score on the cross-platforms datasest.

Figure 9 and Fig. 10 present the results of the component ablation experiments for scRDAN on the cross-platforms dataset, evaluating Accuracy and Silhouette score. Removing adversarial learning (denoising) caused a 1.1% drop in accuracy and a 9.4% decrease in Silhouette score, highlighting the importance of feature reconstruction and adversarial learning in extracting domain-invariant features. Without adversarial training, accuracy decreased by 0.8% and Silhouette score by 11.2%, indicating its role in addressing batch effects. Removing triplet loss led to a 0.8% accuracy drop and a 20.8% decrease in Silhouette score, demonstrating the fine-grained discrimination module’s importance for distinguishing cell types. Excluding consistency loss resulted in a 0.3% accuracy decrease and a 1.2% drop in Silhouette score, suggesting its role in enhancing stability. Finally, removing virtual adversarial training loss caused a 0.7% accuracy drop and a 1.2% Silhouette score decrease, emphasizing its importance in improving robustness and adaptability to noise.

### Hyperparameter sensitivity analysis

**Figure 11:**
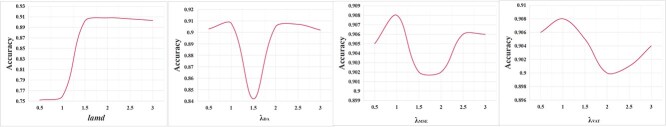
Hyperparameter sensitivity analysis.

A systematic exploration of four key hyperparameters ($lamd$, ${{\lambda } _{DA}}$,${{\lambda }_{MSE}}$, and ${{\lambda }_{VAT}}$) was conducted within the range of 0.5 to 3.0, as shown in Fig. 11. The results demonstrate that the selected parameter combination ($lamd=2$, ${{\lambda }_{DA}}=2$, ${{\lambda }_{MSE}}=1$, ${{\lambda }_{VAT}}=1$) achieves stable and superior performance across different settings, with accuracy reaching or approaching the highest value (0.908). Specifically, $lamd=2$ and ${{\lambda }_{DA}}=2$ effectively balance discriminative power and domain alignment, preventing overfitting and misalignment. ${{\lambda }_{MSE}}=1$ provides sufficient reconstruction constraint, enhancing the robustness of feature representations. ${{\lambda }_{VAT}}=1$ introduces moderate perturbations while maintaining training stability. Overall, this parameter set ensures strong model performance and generalization ability, making it the most reasonable and robust choice.

## Conclusion

In this study, we propose a robust denoising domain adaptation network, scRDAN, to alleivate noise (denoising and noise addition) and distribution alignment consistency (domain global and cell level) in cell type annotation tasks. scRDAN includes three modules: a denoising domain adaptation module, which removes noise and aligns cell features across batches via adversarial learning; a fine-grained discriminative module, which enhances the discriminability of cell types by controlling positional relationships in feature space; and a robustness-enhancement module, which introduces noise in both domains to improve the model’s robustness against dataset variations. Evaluations on various datasets show that scRDAN outperforms other methods.

Despite its effectiveness, scRDAN has some limitations. While it mitigates batch effects through distribution alignment at both the global and cell levels, more flexible alignment strategies that dynamically adjust to data characteristics are needed to better handle batch effects and cell type heterogeneity. These advancements could further enhance the accuracy of cell type annotation.

**Key Points**

- A denoising domain adaptation module is designed, combining denoising and adversarial learning strategies to address noise and data distribution inconsistencies. This approach also achieves precise alignment between source and target domain data, helping to alleviate batch effects and providing dual control over both noise and batch effects, thereby ensuring optimal cell type annotation performance.
- To address blurred cell type boundaries from domain global alignment, a fine-grained discrimination module is introduced. This module captures fine-grained differences between cells and controls positional relationships between similar and different cell types, ensuring clear distinction of adjacent cell types and reducing boundary blurring, thus enhancing cell type annotation.
- Recognizing that noise in single-cell data cannot be completely avoided, a robustness enhancement module is designed. This module introduces various forms of noise to train the model to effectively handle interference, thereby significantly improving overall cell type annotation performance.
- The performance of scRDAN is evaluated on simulated, cross-platforms, and cross-species datasets. Results show that scRDAN outperforms other methods in Accuracy, Silhouette score, and UMAP visualization.

## Data Availability

The code is accessible at https://github.com/CDMBlab/scRDAN.
